# Hormonal predictors of the insulin sensitive phenotype in humans

**DOI:** 10.17305/bb.2025.12210

**Published:** 2025-04-07

**Authors:** Mohamed Badie Ahmed, Abdella M Habib, Saif Badran, Abeer Alsherawi, Sherouk Essam Elnefaily, Mansour Binfayed, Atalla Hammouda, Graeme E Glass, Ibrahem Abdalhakam, Humam Emad Rajha, Abdul-Badi Abou-Samra, Suhail A Doi

**Affiliations:** 1 Department of Population Medicine, College of Medicine, QU Health, Qatar University, Doha, Qatar; 2 Plastic Surgery Department, Hamad General Hospital, Hamad Medical Corporation, Doha, Qatar; 3 Department of Basic Medical Sciences, College of Medicine, QU Health, Qatar University, Doha, Qatar; 4 Division of Plastic & Reconstructive Surgery, Washington University School of Medicine, Saint Louis, MO, USA; 5 College of Medicine, QU Health, Qatar University, Doha, Qatar; 6 Faculty of Pharmacy, Cyprus International University, Nicosia, North Cyprus; 7 Division of Craniofacial Surgery, Department of Plastic Surgery, Great Ormond Street Hospital for Children NHS Foundation Trust, London, UK; 8 Qatar Metabolic Institute, Academic Health System, Hamad Medical Corporation, Doha, Qatar

**Keywords:** Obesity, adipokines, gut hormones, body contouring surgery, insulin sensitivity, pancreatic polypeptide, PP, gastric inhibitory polypeptide, GIP, leptin, liver-expressed antimicrobial peptide 2, LEAP2

## Abstract

Clinical obesity, a chronic condition marked by excessive fat accumulation, often leads to insulin resistance and a heightened risk of comorbidities. This study aimed to identify hormonal predictors of an insulin-sensitive phenotype (ISP) in patients undergoing body contouring surgeries, focusing on the relationship between gut hormones, adipokines, and fat mass. ISP was defined as the highest tertile of HOMA insulin sensitivity. We prospectively followed patients undergoing abdominoplasty, lower body lift, or thigh lift at Hamad General Hospital from January 2021 to December 2023. Body composition, glycemic indices, and hormonal levels were assessed, with data analyzed using descriptive statistics and regression models. The study included 34, 22, and 27 subjects at visits 1, 2, and 3, respectively. Fat percentage decreased slightly at visits 2 and 3 compared to baseline, though not significantly. Median levels of glucagon-like peptide-1 (GLP-1), gastric inhibitory polypeptide (GIP), pancreatic polypeptide (PP), liver-expressed antimicrobial peptide 2 (LEAP2), and amylin varied significantly across visits, initially rising at visit 2 before declining at visit 3. Logistic regression revealed that ISP was negatively associated with serum GIP. LEAP2, and leptin levels while positively associated with PP. History of bariatric surgery was only weakly associated with the ISP after hormonal associations were accounted for. Notably, total body fat percentage did not predict ISP after accounting for hormonal factors. This study highlights GIP, PP, leptin, and LEAP2 as key predictors of ISP, with GIP being the primary negative regulator. These findings underscore the importance of hormonal interplay in insulin sensitivity, emphasizing the role of gut hormones and adipokines in predicting ISP in humans.

## Introduction

Clinical obesity is a chronic health condition characterized by the abnormal accumulation of excess body fat, which negatively impacts overall health and significantly increases the risk of various comorbidities. The expansion of adipose tissue is strongly associated with insulin resistance, a metabolic disorder in which the body’s cells exhibit reduced responsiveness to normal insulin levels, leading to compensatory hyperinsulinemia. Insulin resistance is a key contributor to numerous comorbidities, including metabolic syndrome, which heightens the risk of cardiovascular diseases and metabolic dysfunction–associated steatotic liver disease [1].

Adipose tissue, in addition to being an efficient energy resource, is considered an endocrine organ that secretes various hormones known as adipokines, which play a major role in maintaining metabolic homeostasis. The accumulation of fat tissue that leads to insulin resistance is itself regulated by fat-derived adipokines, which are regulated by less well-known mechanisms. In recent years, key interest has been generated by gut hormones, given their association with weight loss in individuals receiving pharmacotherapy with gut hormone analogs. Dysregulation of gut hormones may lead to dysregulation of adipokines, and among these adipokines, leptin is considered a critical hormone that plays a key role in fat mass regulation [2–4]. It is mainly secreted by adipocytes, and its plasma concentration increases in proportion to body fat mass. Circulating leptin crosses the blood–brain barrier to regulate central nervous system functions, particularly in the hypothalamus [5].

Although leptin has clearly been effective in inducing weight loss and improving insulin sensitivity in leptin-deficient individuals, the same has not been observed in those with leptin excess in lifestyle-related obesity [6]. While leptin excess accrues as fat mass accumulates, the expected effects of leptin on insulin sensitivity, appetite, and other target functions are not seen. It has been suggested that a hormonal co-factor—perhaps also fat-derived and inversely associated with fat mass—may be required in the presence of obesity to prevent leptin resistance [7]. Another hypothesis is that gut hormones may regulate leptin sensitivity, with or without such a co-factor, and may modulate leptin responsiveness [8]. The latter is supported by several observations, including changes in gut hormones following bariatric surgery and weight loss achieved with gut hormone analog therapy [9–12].

Body contouring surgery is a group of procedures in which plastic surgeons aim to surgically remove subcutaneous fat tissue, commonly from the abdomen and thighs, to improve body shape immediately after surgery. These patients present with varying degrees of BMI elevation and/or insulin sensitivity and undergo changes in their subcutaneous fat mass during surgery. Several studies have shown that the metabolic impact of these surgeries may include a decrease in leptin and improvement in insulin sensitivity [7]. However, the underlying mechanisms behind these changes, as well as the impact of these surgeries on incretin and pancreatic hormones, have never been reported [13]. Understanding the relationships between body fat, various hormonal changes, and insulin sensitivity may provide better insight into the mechanisms linking body fat and the insulin-sensitive phenotype (ISP). This may lead to a better understanding of the mechanisms linking clinical obesity to associated metabolic disease and help differentiate it from preclinical obesity, or what is known as insulin-sensitive obese individuals [14]. In the partner paper to this one, we demonstrate a complex relationship between gut hormones (gastric inhibitory polypeptide [GIP], amylin), leptin, and the lean phenotype [15]. In this paper, we examine hormonal predictors of insulin sensitivity to determine whether the same predictors of the lean phenotype also predict the ISP, or if the regulation of insulin sensitivity remains distinct from the regulation of fat mass.

## Materials and methods

### Study population

We studied patients who underwent body contouring surgeries at Hamad General Hospital between January 2021 and December 2023, following them at three key time points: preoperatively, and at 2–3 weeks and 6–10 weeks postoperatively. These time points were selected to capture both immediate and delayed changes in hormonal profiles after surgery.

Eligible participants were adults (≥18 years) with a BMI ≥18 who consented to abdominoplasty, lower body lift, or thigh lift. The study included patients with a history of bariatric surgery (gastric bypass or sleeve gastrectomy), provided the surgery had been performed 18 months or more prior to recruitment. These participants were included to facilitate the examination of the impact on gut hormones. Exclusion criteria included comorbidities (except non-pharmacologically managed diabetes), diabetic nephropathy, contouring outside the abdomen or thighs, age > 65, or BMI > 35. Informed consent was obtained from all participants prior to their inclusion in the study.

### Assessment of body composition

The study assessed subjects’ body composition before and after surgery using the Tanita (DC-360 P) body composition analyzer, which employs bioelectrical impedance analysis (BIA) technology [16]. This device measures multiple variables, including weight, body fat percentage, body fat mass, BMI, fat-free mass, estimated muscle mass, total body water, visceral fat rating (VFR), and basal metabolic rate (BMR). The Tanita analyzer operates by sending low, safe electrical signals through four metal electrodes; these signals pass quickly through hydrated muscles (water) and encounter resistance when passing through fat tissue. The results are processed using scientifically validated Tanita equations to generate a detailed body composition report [17].

### Oral glucose tolerance test (OGTT)

Subjects were required to fast for a minimum of 8 h prior to the assessment. Fasting glucose levels were first measured in the fasting state. Subsequently, a 75-g oral glucose drink was administered, and plasma glucose levels were measured again at 15, 30, 45, 60, and 120 min using a rapid multi-assay analyzer (Analox-GL5).

### Glycemic indices

Homeostasis model assessment was performed for each subject at the defined time points (before and after the surgery) using the University of Oxford HOMA2 calculator, which estimates steady-state beta cell function and insulin sensitivity as percentages of a normal reference population [18]. Samples for C-peptide and glucose were analyzed immediately after collection in the fasted state.

### Body contouring surgeries

Subjects who met the inclusion criteria underwent standard surgical procedures, including abdominoplasty, lower body lift, and/or thigh lift. All surgeries were conducted by expert surgeons in the department (Plastic Surgery, HMC).

### Hormonal measurements

Plasma samples were collected, aliquoted, and stored at –70 ^∘^C until analysis. Levels of GIP, glucagon-like peptide-1 (GLP-1), pancreatic peptide (PP), amylin, and leptin were measured using EMD Millipore’s MILLIPLEX^®^ Human Metabolic Hormone Panel V3, which utilizes Luminex xMAP technology to simultaneously quantify these analytes in human plasma, tissue lysates, and culture supernatants. Additionally, spexin and liver-expressed antimicrobial peptide 2 (LEAP2) were evaluated using ELISA kits from Abbexa Ltd. All samples were analyzed in duplicate within a single assay to minimize inter-assay variability, and the intra-assay coefficient of variation was maintained below 10% to ensure precision. We purchased only seven hormones (GLP-1, PYY, GIP, amylin, leptin, PP, and secretin) out of those available within the multiplex system. These were selected as the most relevant hormones of interest, and we reported only the hormones listed above that contributed to prediction in the model. Spexin and LEAP2 were evaluated using separate kits, as they were not available within the multiplex system.

### Sample size

Sample size calculations were not done because they require knowledge of the true effect in the study, which is always unknown (not only before but also after the study is conducted), and which, if known, would make conducting the study unnecessary [19]. Further, post hoc assessments of power were not done because they are deeply problematic (e.g., they are irrelevant, typically biased, and have large sampling variation) and thus were not calculated [20–23]. Instead, in this paper, we included all participants who were available within the time frame of the study.

### Ethical statement

The study received institutional review board (IRB) approval from Hamad Medical Corporation under reference MRC-01-20-466.

### Statistical analysis

Descriptive statistics for patient demographics and variables of interest were reported at each time point (preoperative and postoperative). Differences between these time points were calculated for each variable. Trends in hormonal parameters of interest were evaluated using regression models, with visits (1–3), bariatric surgery status (RYGB, SG, or none), and demographic characteristics included as covariates. Logistic regression models were used to predict the upper tertile of insulin sensitivity, which was labeled the ISP. The correlational structure of repeated measurements in the same patient over time was addressed using cluster-robust standard errors. To better understand the relationships, a margins plot was created to depict the results indicated by logistic regression.

To determine if the study data were consistent with a population model (tested hypothesis) that assumes no effect, a *P* value was computed [24]. The exact *P* value was reported and indicated the degree of divergence of the estimated effect from the null hypothesis, had it been the source of the study data. Results in the interval *P* < 0.05 were labeled “statistically divergent” [24]. To assess clinical benefit, the point estimate and its 95% uncertainty interval (95% UI) were reported, which enables an assessment of the practical importance of the study result. All analyses were conducted using Stata Version 17 (StataCorp, College Station, TX, USA).

## Results

### Participants’ characteristics

At visits 1, 2, and 3, the number of subjects who attended was 34, 22, and 27, respectively. BMI, fat percentage (Tanita), history of bariatric surgery, and median hormone levels (leptin, spexin, GLP-1, GIP, PP, amylin, and LEAP2) were compared across the three visits (Table S1). The gender distribution remained consistent throughout the visits, with females constituting the majority at each time point. BMI values showed minor fluctuations across visits, with no practically important differences observed. Fat percentage decreased at visits 2 and 3 compared to baseline, though these changes were not statistically divergent. Similarly, the history of bariatric surgery did not show statistically divergent variations across the three visits (Table S1).

**Table 1:** Predictors of ISP using logistic regression

**ISP**	**OR**	***P* > |z|**	**95% uncertainty interval**
*Visit*			
1	1 (base)		
2	1.549	0.573	0.339, 7.088
3	0.442	0.211	0.123, 1.591
*History of bariatric surgery*			
No	1 (base)		
BP	1.483	0.682	0.226, 9.748
SL	2.900	0.754	0.822, 10.226
PP (pg/mL)	1.005	0.116	0.999, 1.010
GIP (pg/mL)	0.993	**0.009**	0.987, 0.998
Leptin (ug/L)	0.944	0.073	0.886, 1.006
LEAP2 (pg/mL)	0.879	0.221	0.715, 1.081
Constant (baseline odds)	1.968	0.484	0.296, 13.65

*Goodness of fit AUC ═ 0.77; McFaddens *R*^2^ ═ 0.182; goodness of link ascertained via linktest in Stata. The model was adjusted for time as some samples were taken 15 min after glucose load as well as body fat percentage. However, body fat percentage was not predictive (OR ═ 0.965, *P* ═ 0.409) and was removed from the final model. The 15-min time points refer only to gut hormones where some samples did not have time 0 available. All HOMA calculations were from fasting samples only (see methods). ISP: Insulin-sensitive phenotype; PP: Pancreatic polypeptide; GIP: Gastric inhibitory polypeptide; LEAP2: Liver-expressed antimicrobial peptide 2.

Median levels of leptin and spexin fluctuated across visits. Spexin exhibited a consistent upward trend, while leptin levels decreased slightly at visit 2 before rising again at visit 3; however, these changes were not statistically divergent. In contrast, GLP-1, GIP, PP, LEAP2, and amylin levels displayed statistically divergent variations across visits, with an initial increase at visit 2 followed by a decline at visit 3, indicating notable temporal changes.

### Predictors of the ISP

**Figure 1.**
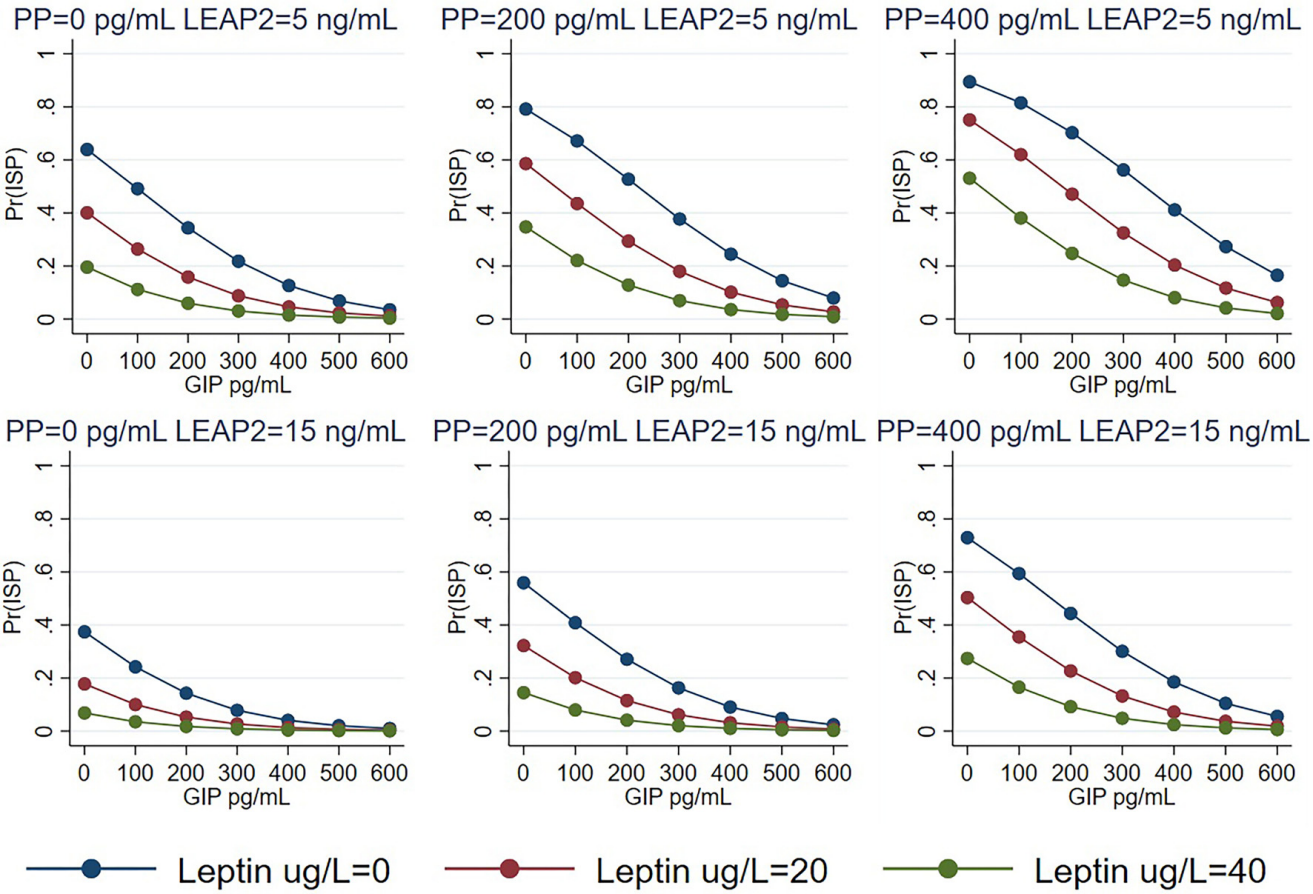
**Margins plot with the probability of having ISP as a function of GIP level across three plasma leptin (µg/L) levels, two LEAP2 (ng/mL) levels (5 and 15) and three PP (pg/mL) levels (0, 200, 400).** These plots derive from the logistic regression model shown in Table 1. ISP: Insulin-sensitive phenotype; PP: Pancreatic polypeptide; GIP: Gastric inhibitory polypeptide; LEAP2: Liver-expressed antimicrobial peptide 2.

When predictors of ISP were analyzed using logistic regression (Table 1), there was a negative association of ISP with plasma LEAP2, leptin, and GIP levels, with the latter demonstrating statistically divergent results. On the other hand, there was a positive association of ISP with PP and history of bariatric surgery—more so for sleeve gastrectomy—after accounting for the impact of leptin and gut hormones. A margins plot demonstrated that the probability of ISP was mainly predicted by GIP, with the other hormones modulating this relationship across GIP levels (Figure 1).

An additional analysis was conducted to include total body fat percentage (Tanita) within our regression model for predicting ISP and, surprisingly, it was not predictive within the model after the other hormones were factored in (fat percentage: OR ═ 0.965, *P* ═ 0.409). This finding is consistent with the observation that increasing fat mass is associated with a pattern of hormonal changes that predict ISP.

## Discussion

Our results highlight the complex relationship between LEAP2, PP, leptin, and GIP on the ISP. The findings showed that GIP is a key player in predicting ISP, the probability of which declines as GIP increases. In addition, increases in LEAP2 and leptin were negatively associated with the insulin-sensitizing effect of low GIP, while PP was positively associated with the insulin-sensitizing effect of low GIP. Although our findings show associations, we cannot conclusively determine a cause-and-effect relationship between plasma hormone levels and ISP prediction. However, the existing literature presents strong evidence suggesting that these hormones influence the ISP in these individuals.

It is well known that GIP is an incretin hormone and therefore stimulates pancreatic secretion of insulin [25, 26]. GIP is associated with insulin resistance, as its levels increase, as shown by our results, and therefore the increase in insulin secretion may also be, at least in part, secondary to increases in insulin resistance. This finding aligns with existing data in the literature, as evidenced by a study conducted on mouse models, which showed that administration of a GIP receptor antagonist ((Pro3) GIP) in ob/ob mice led to a significant improvement in insulin sensitivity, independent of any changes in food intake or body weight. The (Pro3) GIP-treated group also exhibited a significant reduction in pancreatic insulin content and partial amelioration of islet hypertrophy and β-cell hyperplasia [27]. Another study assessed the effect of a high-fat diet on wild-type mice and mice lacking GIP receptors [28]. The wild-type mice developed GIP hypersecretion, extreme visceral and subcutaneous fat deposition, and insulin resistance. In contrast, the mice lacking GIP receptors were protected from obesity and insulin resistance. Furthermore, the effects of (Pro3) GIP injections were investigated in high-fat diet-fed mice over a 160-day period [29]. The results demonstrated that GIP antagonism for 50 days significantly improved insulin sensitivity and facilitated the reversal of glucose intolerance and diabetes. These findings collectively suggest that increasing levels of GIP contribute to insulin resistance as well as excessive insulin secretion (hyperinsulinemia) and β-cell hyperplasia, making it a promising therapeutic target for improving insulin sensitivity and managing metabolic disorders such as type 2 diabetes.

Our data suggest that PP augments the effect of lower levels of GIP on ISP and, thereby, should decrease insulin secretion from the pancreas as insulin sensitivity improves with increasing PP levels. This aligns with the body of knowledge in the literature, as PP has been reported to play a role in feeding, body weight, and energy balance [30, 31]. In a rodent study, PP-sterically stabilized micelles (SSM) improved glucose tolerance and insulin sensitivity in rats with pancreatogenic diabetes caused by pancreatic diseases like chronic pancreatitis and pancreatic neoplasia [32]. Similarly, PP infusion in patients with type 1 or pancreatogenic diabetes on insulin pump therapy enhanced insulin sensitivity and reduced the insulin dose needed to maintain normal glucose levels [33]. A study in obese children found that, at baseline, they had higher insulin resistance, elevated leptin, and lower PP levels compared to normal-weight children [34]. After one year of weight loss, the obese group showed increased PP levels, which correlated with reduced leptin levels and improved insulin sensitivity. Similar findings were reported in a study that assessed PP, insulin sensitivity, and DPP-IV in obese children over one year of a weight loss intervention program. The results showed a significant increase in PP and insulin sensitivity, with a significant decrease in DPP-IV in children with substantial weight loss [35]. Although we demonstrate that the effect of PP on the ISP opposes that of GIP, several studies have shown that PP also increases as a consequence of increased GIP. One study investigated the effect of human GIP1-42 (hGIP) administration on PP levels. The results revealed that hGIP significantly increases PP secretion in healthy individuals, patients with type 2 diabetes, and isolated porcine pancreata [36]. Additionally, another study examined the impact of hGIP injection on PP secretion in overweight/obese individuals with type 2 diabetes mellitus who were undergoing treatment with metformin and a long-acting GLP-1 receptor agonist [37]. The findings indicated that PP concentrations during GIP infusion were significantly higher compared to those during placebo infusion at all measured time points. These results suggest that PP could be responding to GIP receptor activation—the latter inducing a state of insulin resistance—and that, through unknown mechanisms, PP secretion increases to counter this effect. This was not mediated through GLP-1, as it is known that GLP-1 does not stimulate PP secretion [38]. Collectively, these studies underscore the intricate interplay between GIP, PP, and insulin sensitivity, highlighting the importance of further research to better understand these relationships.

Leptin was associated with an increase in insulin resistance as its levels increased. The leptin effect is paradoxical, as it is well known to be an insulin sensitizer, but this finding potentially represents ongoing stimulation of leptin release in a state of leptin resistance [39]. We have reported in our partner paper in this journal that leptin levels increase primarily with fat mass and, therefore, high leptin is itself a proxy for leptin resistance [15]. This likely explains why high leptin was associated with greater insulin resistance in this study. Elevated leptin levels, driven by increased fat mass, are associated with both leptin resistance and insulin resistance [15]. This suggests that leptin resistance contributes to impaired insulin sensitivity and lowers the probability of the ISP.

LEAP2 has been shown to be associated with glucose homeostasis and body weight in both human and mouse models. In diet-induced obesity (DIO) mice, LEAP2 levels were significantly higher compared to the control group and were positively correlated with fat mass and body weight [40]. In humans, BMI, body fat percentage, and HOMA-IR showed a positive correlation with LEAP2 levels even after adjusting for age and sex [41]. In another clinical trial that assessed a cohort with prediabetes and overweight/obesity, fasting plasma LEAP2 levels were inversely associated with insulin sensitivity and positively associated with BMI, body weight, and fat mass [42]. The accumulating data therefore indicate that LEAP2, similar to leptin resistance, is negatively associated with insulin sensitivity, aligning with our findings.

Finally, the strength of this study lies in a clear analytical plan that is able to correctly model the observed relationships. However, this study is limited by a moderate sample size and may benefit from replication by future researchers. Nevertheless, as indicated in the footnote in Table 1, the goodness of fit of the model was quite good despite the moderate sample size.

## Conclusion

**Figure 2.**
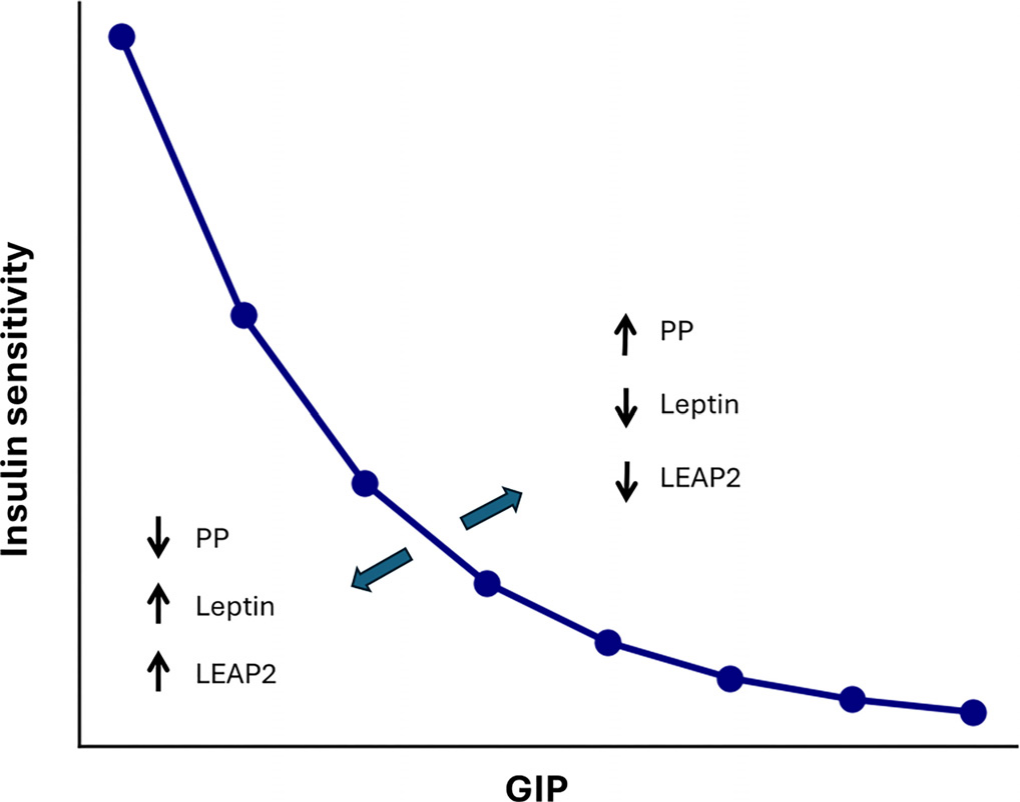
**This diagram illustrates the relationship between GIP levels and insulin sensitivity, highlighting key factors that modulate this relationship based on our findings.** PP: Pancreatic polypeptide; GIP: Gastric inhibitory polypeptide; LEAP2: Liver-expressed antimicrobial peptide 2.

This study demonstrates, for the first time, that GIP, PP, leptin, and LEAP2 are predictors of ISP in humans. A strong negative relationship exists between plasma GIP and ISP, which is further modulated by PP, LEAP2, and leptin. It can therefore be concluded that ISP is modulated by gut hormones, which also modulate body fat. This challenges the conventional wisdom that body fat is the main regulator of ISP. Figure 2 illustrates these findings and raises key questions for future research, particularly regarding the mechanisms and pathways underlying this relationship, and whether the effects are direct or indirect.

## Supplemental data

Supplemental data are available at the following link:

https://www.bjbms.org/ojs/index.php/bjbms/article/view/12210/3856
